# Chemogenetic Modulation of Astrocytic Activity Rescues Hippocampus Associated Neurodegeneration in Alzheimer's Disease Mice Model 5xFAD

**DOI:** 10.1155/np/9880933

**Published:** 2025-10-06

**Authors:** Evgenii Gerasimov, Maria Berg, Anastasia Bolshakova, Ilya Bezprozvanny, Olga Vlasova

**Affiliations:** ^1^Laboratory of Molecular Neurodegeneration, Institute of Biomedical Systems and Biotechnology, Peter the Great St. Petersburg Polytechnic University, St. Petersburg 195251, Russia; ^2^Laboratory of Molecular Neurobiology, Pavlov Institute of Physiology, Russian Academy of Sciences, St. Petersburg 199034, Russia

**Keywords:** 5xFAD, β-amyloid, Alzheimer's disease, astrocytes, chemogenetics, CNO, DREADDs, GFAP, hM3D

## Abstract

Alzheimer's disease (AD) is a prevalent neurodegenerative disorder characterized by Aβ-amyloid accumulation and cognitive decline. Despite extensive research, effective treatments remain elusive. Astrocytes, the most abundant glial cells, play a crucial role in synaptic transmission, neuronal excitability, and plasticity. In AD, astrocytes become reactive, exhibiting aberrant calcium signaling and altered neurotransmitter release, making them promising targets for disease-modifying therapies. To address this, we explored designer receptors exclusively activated by designer drugs (DREADDs), specifically the hM3D(Gq) receptor, which selectively modulates intracellular Ca^2+^ levels in astrocytes upon activation by clozapine-N-oxide (CNO). Using daily CNO administration in 8-month-old 5xFAD mice, we observed a significant enhancement of impaired long-term potentiation formation, accompanied by cognitive improvements in the fear conditioning (FC) and Morris water maze (MWM) tests. Additionally, anxiety levels and social preference deficits in 5xFAD mice were fully restored following astrocytic activity modulation. Importantly, this approach reduced Aβ-amyloid plaque burden and demonstrated a trend toward mitigating astrocytic reactivity, further highlighting its therapeutic potential. Our findings suggest that targeting astrocytic activity via Gq-coupled receptors represents a novel and promising strategy for AD treatment, offering a noninvasive and effective approach to mitigating disease progression.

## 1. Introduction

Alzheimer's disease (AD) is one of the most widespread neurodegenerative diseases nowadays. Its pathogenesis is associated with toxic Aβ-amyloid accumulation, which is formed by aberrant γ-secretase activity [1–3]. Moreover, AD progression is also accompanied by tau tangles accumulation [4, 5], neuronal death [6], and significant cognitive decline [7, 8]. However, there is no reliable cure for this type of neurodegeneration [9, 10]. Most of the therapeutic interventions were aimed at Aβ-amyloid clearance from the brain, albeit they were not successful enough [11]. Many approaches were also targeted at neuro- and synaptic protection, which were based on the neuronal prevention from Aβ-amyloid toxicity and excitotoxicity [12–14]. These types of therapies were focused only on neurons, leaving other cell types in the brain out of focus. In the last decade, numerous studies have focused on astrocytes—electrically nonexcitable nerve cells which are indispensable participants in the tripartite synapse [15–20]. Astrocytes are one of the most abundant cells in the brain, playing a pivotal role in the regulation of neuronal function, such as excitability [21, 22], synaptic transmission [23], and plasticity [23–25]. These cells undergo pathophysiological changes during AD progression [26–29], making them promising targets for disease-modifying treatments [30, 31]. In AD, astrocytes transform into a reactive form, where aberrant calcium signaling, neurotransmitter release, and metabolism are observed [32, 33]. Thus, considering the above stated, recovering impaired astrocytic functioning during AD is a promising avenue for possible therapy.

In our recent studies, we have demonstrated a method for manipulating astrocytic activity using optogenetics. Activation of OPTO-α1AR opsin (a Gq-coupled pathway involvement), expressed in the hippocampal astrocytes, led to elevation of spontaneous excitatory postsynaptic currents (sEPSCs) in the pyramidal neurons with facilitation of the field excitatory postsynaptic potentials (fEPSPs) in this area with a beneficial impact on a long-term potentiation formation, mushroom spines preservation, and cognitive functions in the 5xFAD mice [34]. However, this approach imposes critical issues such as optical fiber implantation, thus, lowering its translational value [35]. To overcome these limitations in the current manuscript, we performed experiments involving designer receptors exclusively activated by designer drugs (DREADDs), specifically hM3D(Gq) [36, 37]. This type of receptors does not have any natural agonists in the mammals and are only activated via clozapine-N-oxide (CNO). HM3D activation leads to IP3-receptor activation and elevation of Ca^2+^ in the cytoplasm of the astrocytes [38]. We used the hM3D(Gq) receptor that was selectively expressed in astrocytes by performing bilateral injections into the dorsal hippocampus of mice, utilizing viral vectors driven by the astrocyte-specific glial fibrillary acidic protein (GFAP) promoter. The hippocampus is one of the brain regions most affected by AD, exhibiting significant synaptic loss and extensive amyloid plaque deposition [39]. Additionally, memory decline is closely associated with hippocampal dysfunction [40]. For these reasons, this region was selected for targeted astrocytic activity modulation.

Daily intraperitoneal (IP) CNO injections of 8-month-old 5xFAD mice model of AD [41–43] had a positive effect on their cognitive functions in behavioral tests such as fear conditioning (FC) and Morris water maze (MWM). These beneficial changes were also confirmed using ex vivo probing of synaptic strength by long-term potentiation recordings, where repeated hippocampal astrocytes activity modulation restores altered LTP formation in 5xFAD transgenic mice. Moreover, we have observed altered anxiety levels and social preference of control transgenic 5xFAD mice, which were totally recovered after sequential IP injections of CNO in experimental group of 5xFAD mice. Everyday modulation of hippocampal astrocytes significantly reduced β-amyloid positive area and demonstrated a trend toward mitigating astrocytic reactivity. Thus, in this manuscript a novel promising disease-modifying approach for AD through astrocytic activity modulation using exogenous Gq-coupled receptors is suggested.

## 2. Materials and Methods

### 2.1. Animals

5xFAD mice (Jackson Laboratory, USA; strain #034848) on a C57BL/6 background were used in this study. Both male and female mice, aged 8 months, were included in the experimental groups. Male and female mice were kept separately. 5xFAD mice aged 8 months demonstrated significant memory decline, synaptic failure, and severe Aβ-amyloid deposition [41], so this age was chosen to confirm efficiency and reliability of astrocytic activity modulation approach as disease-modifying strategy for AD. The breeding colonies were established and maintained in the vivarium of the Laboratory of Molecular Neurodegeneration at Peter the Great St. Petersburg Polytechnic University. Mice were housed in groups of 4–6 per cage under a 12-h light/dark cycle with ad libitum access to food and water.

### 2.2. Viral Constructs Delivery

All surgeries were performed under 1.5%–2.0% isoflurane anesthesia. Surgical procedures proceeded only if the mouse did not respond to a single paw pinch. Stereotaxic surgery (68001, RWD Life Science, China) was performed using a heated mat with a temperature controller (69002, RWD Life Science, China) set to maintain a constant temperature of 37°C. Control mice groups (WT + veh and 5xFAD + veh) received bilateral injections of PBS, while experimental mice groups (WT + hM3D and 5xFAD + hM3D)—AAV5–GFAP–hM3D(Gq)–mCherry (#50478-AAV5; final titer >7 × 10^12^ vg/mL) at the following coordinates: AP −2.1 mm, DV −1.45 mm, ML ±1.4 mm into dorsal hippocampal region. A total volume of 1.05 µL of the virus was infused at a rate of 0.1 µL per minute using a Hamilton syringe (#84853, 7758-02, Hamilton, USA). After surgery, mice were kept on a heating mat and supplied with oxygen for 3–5 min until they were able to walk normally, after which they were transferred to their home cages.

### 2.3. IP Injections of CNO

A 3 mg/kg of CNO (#34233-69-7, LEAP Chem, China) diluted in saline (0.5% of DMSO) were IP injected (IP started as mice aged 7.5 months old) for 2 weeks before, each day during behavioral tests and long-term potentiation experiments. All mice groups (WT + veh, WT + hM3D, 5xFAD + veh, 5xFAD + hM3D) underwent daily injections of CNO. A 2-week period of IP CNO injections prior to behavioral testing was chosen, as this duration is sufficient to induce molecular and cellular changes, and is consistent with previously published studies [44–47]. Before and during the behavioral testing period, mice had received a total of 31 IP injections of CNO, administered daily at the same time (10:30 AM). Behavioral testing began 2 h after injections. When the tests ended, on the following day, one group of mice was perfused for fixed tissue slice preparation, while the other group continued to receive CNO injections during long-term potentiation experiments.

### 2.4. FC

Behavioral testing started when mice achieved 8 months old. Day 1: The mouse was placed in Cage A for 5 min, allowing free exploration without exposure to any conditioned or unconditioned stimuli (e.g., sound or shock). The insulation cabin door remained tightly closed throughout the session. Afterward, the mouse was returned to its home cage. Day 2: The mouse was placed in Cage A for 2 min. A conditioned stimulus (80 dB sound and 10 kHz) was presented for 20 s, followed by an unconditioned stimulus (500 µA shock) lasting 1.5–2 s. This sequence was repeated once, followed by a 1-min rest period before the mouse was returned to its home cage. Day 3 (early long-term memory test): The mouse was reintroduced to Cage A for 3 min to assess contextual fear memory based on its prior negative experience. The test cage was then modified to create a new, unfamiliar environment (Cage B) by adding an extra light source, altering the chamber walls, and introducing a vanilla scent. The mouse was given 3 min to assess its freezing behavior as a measure of its initial fear response (Pretone phase). Next, the conditioned sound was turned on (during tone phase) and cued memory was recorded [48]. Day 10 (long-term memory test): 1 week later, the same testing paradigm was repeated to evaluate mice′ long-term memory.

To analyze the obtained data, the following protocol was applied: A paired *t*-test was used to compare freezing levels between baseline and context conditions, as well as between pretone and during-tone conditions. If freezing levels during the context or during-tone phases were significantly higher than the corresponding baseline or pretone levels across all groups, further statistical tests (two-way ANOVA with Tukey post hoc test) for multiple comparisons were conducted. These multiple comparisons were used to assess differences in freezing levels during the context phase of the FC test between the experimental groups at the corresponding stage.

### 2.5. Open Field (OF) Behavioral Test

The OF test was used to assess anxiety levels in mice. The OF arena consisted of an opaque plexiglass round chamber with a 62.4 cm diameter, divided into three zones: central (28.4 cm diameter), intermediate, and border (8.5 cm from the chamber wall). The arena was brightly illuminated (950 lux) from above [49]. During the test, each mouse was placed in the center of the arena and its behavior was recorded for 10 min. The arena was cleaned with 70% ethanol after each trial to prevent scent cues. Each mouse underwent a single trial and results were analyzed using Any-maze software (Stoelting Europe, Ireland).

### 2.6. Social and Nonsocial Recognition Behavioral Test

The tests were conducted in the same OF arena. A nonsocial object and a social object (a mouse of the same sex and strain) were placed at the center of the arena. On Day 1, mice were allowed to explore the nonsocial object. On Day 2, their interaction with the social object was assessed. The OF arena was thoroughly cleaned with 70% ethanol after each trial to eliminate scent cues. This type of social and nonsocial test preference paradigm [50, 51] was chosen, as placing nonsocial and social object in the middle of the arena helps researchers to reveal alterations in anxiety and interest level while avoiding possible spatial constraints, mimicking the open spaces interactions.

### 2.7. MWM Test Behavioral Test

The MWM test was conducted in a round white pool (1.50 m diameter, 0.66 m height, and 0.29 m water depth) filled with nontransparent water (due to dissolved dry milk) maintained at 21–23°C [52]. A round hidden platform (0.10 m diameter and 0.28 m height) was placed 1.5 cm below the water surface. Mice underwent four consecutive training days, with four trials per day, to find the hidden platform. To navigate, they relied on four black-and-white visual cues positioned 10 cm above the water surface in different directions. Each trial began from a randomly assigned cardinal direction, with all mice starting from the same positions. They had 90 s to find the platform. If unsuccessful, they were guided to the platform and allowed to rest for at least 15 s. Between trials, mice were placed in heated cages for 20 min to dry. On Day 6 (final day), mice underwent a single 90-s trial without the platform to assess memory properties. The entire test was recorded using a VS 1304-1 video system with Gigabit Ethernet software and performance was analyzed using Any-maze software v7.3, which measured the number of successful trials and average platform search latency during sessions.

### 2.8. Long-Term Potentiation Recordings

After behavioral testing (mice aged 8.5 months) mice were taken for long-term potentiation experiments for analyzing synaptic plasticity in hippocampus. Transcardial perfusion was performed with saturated carbogen (95% O_2_/5% CO_2_) 0–2°C normal ACSF solution-1 (124 mM NaCl, 2.5 mM KCl, 1.2 mM NaH_2_PO_4_, 24 mM NaHCO_3_, 5 mM HEPES, 12.5 mM D-glucose, 0.5 mM CaCl_2_, 10 mM MgSO_4_, and 3 mM Na-pyruvate) and decapitation was performed. Horizontal slices of the brain with a thickness 400 µm for recording of extracellular field potentials were obtained by microtome (Leica VT1200S [Leica Bio-systems Division of Leica Microsystems Inc., Buffalo Grove, IL, USA]) from 8-month-old mice in a 0–2°C solution of normal ACSF solution-1 with carbogen saturation. Then, slices were transferred to a chamber for 30 min incubation in normal ACSF solution-1 at 32°C. Further, acute hippocampal slices were transferred to normal ACSF solution-2 (124 mM NaCl, 2.5 mM KCl, 1.2 mM NaH_2_PO_4_, 24 mM NaHCO_3_, 5 mM HEPES, 12.5 mM D-glucose, 2 mM CaCl_2_, and 0.8 mM MgSO_4_), saturated with carbogen with at a room temperature. All the reagents mentioned above are from Sigma–Aldrich, St. Louis, MO, USA. After an hour after incubation at room temperature, hippocampal slices were used for long-term potentiation experiments. FEPSP were recorded by the glass pipette electrode containing normal ACSF solution-2 with resistance 180–250 kΩ in the CA1 area of stratum radiatum. During recording, fEPSP were filtered at 400 HZ filter with 100 gain value. FEPSP for basal level were recorded at least for 20 min and normalized baseline values did not exceed 20% deviation from mean value. For conditioning protocol high frequency stimulation (HFS) was applied with (100 pulses for 1 s with a two-time repetition after 20 s). Then 60 min potentiated fEPSP were recorded. Only 2–3 recordings per mice were used for LTP recordings analysis.

### 2.9. Fixed-Slice Preparation

After behavioral testing (mice aged 8.5 months) fixed slices were obtained for immunolabelling and Thioflavin-T (Thio-T) staining. Mice were initially anesthetized with urethane (150 mg/mL) and rometar (32 µL/mL), both diluted in 0.9% NaCl. Once the mice exhibited no response to a paw pinch, a thoracotomy was performed to access the heart. A needle was inserted into the left ventricle and a fixative solution (1.5% PFA in PBS, pH 7.4; VWR E404-200 TABS, USA) was perfused for 5–7 min. Following perfusion, the mice were decapitated, and the brains were extracted and placed in 4% PFA for 1 week. To obtain 50 µm sagittal hippocampal slices, a Campden 5100 mz Microtome (Campden Instruments, England) was used. The slices were stored in 0.5% PFA until immunohistochemical experiments. For microscopy analysis, the slices were mounted on microscope slides (Heinz Herenz, #1042050, Germany) using Aqua Poly/Mount (Polysciences, #18606, USA) and covered with coverslips (Menzel Gläser, #1, Germany).

### 2.10. Aβ-Amyloid Plaques Area and Size Analysis

To assess Aβ-amyloid presence in the hippocampus, Thio-T staining was performed. Tissue slices were first placed in PBS for 2 min, followed by incubation in a 1% aqueous solution of Thio-T (T3516, Sigma–Aldrich, USA) for 3 min. Next, the slices were treated with 1% acetic acid for 10 min, then, washed three times in PBS for 4 min each. After staining, the slices were mounted on microscope slides for imaging. Confocal imaging of Aβ-amyloid plaques was conducted using a ThorLabs confocal microscope, equipped with a 4× Olympus objective (Plan N, UIS 2, OLYMPUS, Japan). Imaging parameters were as follows: gain set to 40, input range of 500 mV, field size of 100, resolution of 1.156 µm/pixel, pinhole size of 25 µm, and frame averaging of 9. Image analysis was performed using ImageJ [53] software with the LABKIT plugin. From each mouse, four confocal images were obtained (both hippocampal areas from two fixed slices). Then, they were analyzed and results were averaged.

### 2.11. Immunohistochemical Staining

To visualize hippocampal astrocytes, GFAP immunostaining was performed. Tissue slices were first washed once in PBS for 5 min, followed by incubation in 2N hydrochloric acid for 15 min at room temperature. After acid treatment, slices were washed three times in PBS for 5 min each. Next, slices were incubated in 10% BSA (A9430, Sigma Aldrich, USA) with 0.25% TRITON X-100 (Am-O694-0.1, Helicon, Russia) for 1 h to block nonspecific binding. After blocking, slices were washed once in PBS for 5 min and incubated overnight at 4°C with 1:1000 anti-GFAP antibodies (anti-rabbit; #840001, BioLegend, USA) diluted in 5% BSA with 0.125% TRITON X-100. Following primary antibody incubation, slices were washed three times in PBS for 5 min each and incubated with 1:1500 Alexa Fluor 594 (rabbit; R37117, Invitrogen, USA) diluted in 5% BSA with 0.125% TRITON X-100 for 1 h at room temperature. After secondary antibody staining, slices were washed once in PBS for 5 min and mounted on microscope slides for imaging. Imaging parameters were as follows: gain set to 50, input range of 200 mV, field size of 110, resolution of 1.272 µm/pixel, pinhole size of 125 µm, and frame averaging of 9. Image analysis was performed using ImageJ software. From each mouse, four confocal images were obtained (both hippocampal areas from two fixed slices). Then, they were analyzed and results were averaged.

### 2.12. GFAP Positive Area and Thresholded Size of Single GFAP Positive Unit Analysis

Image analysis was performed using ImageJ software. First, the image sequence obtained from the confocal microscope was averaged to generate a single projection (average intensity). Then, a bandpass filter was applied with the following parameters: filter large structures down to 40 pixels, filter small structures up to 3 pixels, and tolerance direction 5%. Thresholding was performed using the “intermodes” method to produce a binary image. After, “analyze particles” function in ImageJ was used to quantify the GFAP + area parameter and find all the thresholded single GFAP + units (Figure S1). GFAP + area parameter (%) value was computed for each hippocampal image (both hippocampus from two different slices per mice) and then averaged per mice. All thresholded single GFAP + units size (µm^2^) were calculated per individual hippocampus (both hippocampus from two different slices per mice) and then averaged per mice.

### 2.13. Statistics

First, outliers were examined and excluded from analysis prior to any statistical analysis. Outliers were defined as values lower than *Q*_1_ − 1.5 × interquartile range or higher than *Q*_3_ + 1.5 × interquartile range. Then, data normality was assessed using the Shapiro–Wilk or Kolmogorov–Smirnov test. For samples with a normal distribution, Bartlett's test was applied to confirm homogeneity. Based on these test results, comparisons were made using the Student's *t*-test (for parametrically distributed values) or Mann–Whitney test for paired analyses (for nonparametrically distributed values), and two-way ANOVA followed by Tukey's test (for parametrically distributed values), Kruskal–Wallis test followed by Dunn's test, or Conover–Iman test (for nonparametrically distributed values) for multiple comparisons. For two-way ANOVA in the current manuscript the same factors were applied in all comparisons (column factor-genotype [WT or transgenic 5xFAD], row factor-bilateral injections of hM3D-mCherry or vehicle). Statistical significance was set at *p*  < 0.05. All data in figures and text is presented as mean ± standard deviation (SD) if other not mentioned.

## 3. Results

### 3.1. Daily Modulation of Hippocampal Astrocytic Activity Led to Cognitive Functions Recovery in 5xFAD Mice

**Figure 1:**
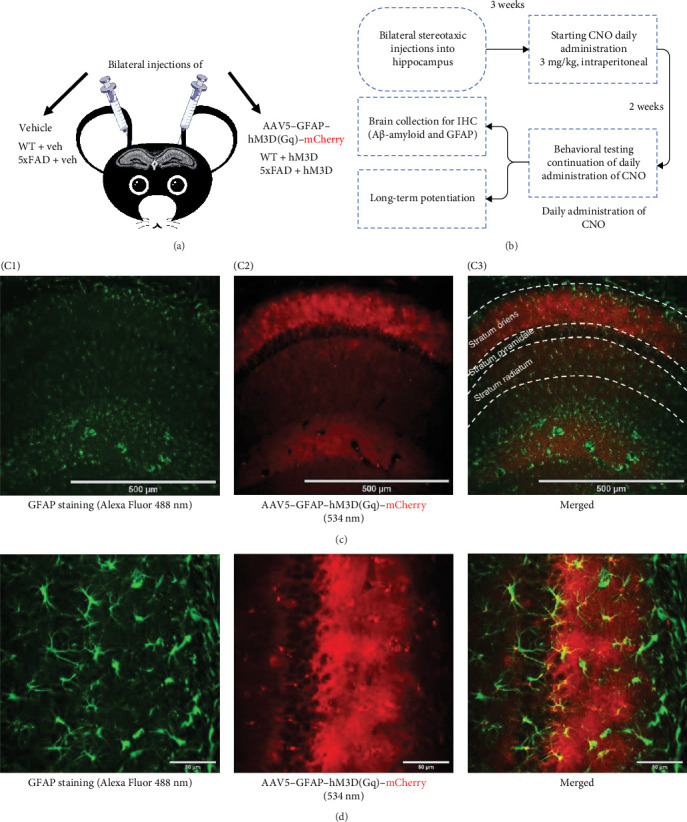
Principal scheme of the experiments. (A) Control and experimental groups of WT and transgenic 5xFAD mice aged 8 months. (B) Schematic illustration of the experimental pipeline. (C, C1) GFAP immunohistochemical staining of mice hippocampus (green); (C2) fluorescence of mCherry (red) fused to hM3D receptor, expressed in the hippocampal astrocytes; (C3) merged confocal images of GFAP-staining and mCherry fluorescence, 10 × magnification, scale bar corresponds to 500 µm. (D) CA1 area of mice dorsal hippocampus with astrocytes expressing hM3D-mCherry, 10 × magnification, scale bar corresponds to 50 µm.

Memory and cognitive decline are among the hallmarks of AD [2, 54]. It has been widely shown that mouse models of AD exhibit cognitive deficits in various cognitive tests [48, 55–57]. To investigate whether modulation of astrocytic activity in the hippocampus might have a beneficial impact on cognitive functions of AD mice model 5xFAD, bilateral injections of AAV5–GFAP–hM3D(Gq)–mCherry into hippocampal region of mice were performed for experimental groups of WT and transgenic 5xFAD mice (WT + hM3D and 5xFAD + hM3D correspondingly), while bilateral injections of vehicle were done for control mice groups (WT + veh and 5xFAD + veh; Figure 1A,C). Viral constructs were delivered to dorsal hippocampus area and expression of mCherry fluorescent protein was observed only in stratum oriens region. It is important to note that a distinctive feature of astrocytes is their highly branched processes, yet GFAP immunolabelling typically reveals only 10%–15% of the astrocytic surface area [58]. In contrast, fluorescent protein labeling, such as mCherry, allows for visualization of finer and more distal processes. Consequently, both GFAP staining and mCherry expression are predominantly co-localized to the soma and proximal branches of astrocytes (Figure 1C,D). After 3 weeks, all mice were administered daily IP injections of 3 mg/kg CNO (Figure 1B).

**Figure 2:**
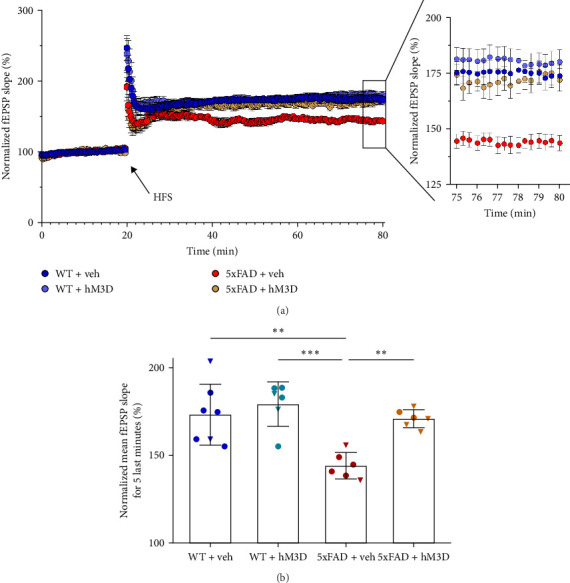
Daily modulation of astrocytic activity in hippocampus led to restoration of altered synaptic plasticity in transgenic 5xFAD mice aged 8 months. (A) The normalized average value of fEPSP value (%) in all mice groups. Via an arrow moment of HFS is demonstrated. In the right corner, last 5 min of an hour recording after potentiation is shown. (B) The normalized mean fEPSP value in the last 5 min averaged per mice (WT + veh: *n* = 7 mice [5♂ and 2♀]; WT + hM3D: *n* = 6 mice [4♂ and 2♀]; 5xFAD + veh: *n* = 6 mice [4♂ and 2♀]; 5xFAD + hM3D: *n* = 6 mice [4♂ and 2♀]). Multiple comparisons between mice groups are done using two-way ANOVA with Tukey post hoc test (*⁣*^*∗∗*^: *M*, *⁣*^*∗∗∗*^: *p*  < 0.001). In graph of Part (B), male mice are represented by circles, and female mice by triangles. Data in Part (A) is presented as mean ± SEM, while data in Part (B) is presented as mean ± SD.

In the first step, we validated the potential of daily hippocampal astrocytic modulation to positively impact synaptic plasticity. We performed long-term potentiation recording—a proxy for a synaptic strength in the acute hippocampal slices—a proxy for a synaptic strength in the acute hippocampal slices [59]. Prior studies have shown that 5xFAD transgenic mice exhibit impaired synaptic plasticity, characterized by reduced LTP formation [60–62]. In this study, acute hippocampal slices were obtained from 8-month-old mice, that had undergone sequential modulation of hippocampal astrocytes (WT + hM3D and 5xFAD + hM3D) and their respective control groups (WT + veh and 5xFAD + veh). To induce long-term synaptic changes HFS protocol was applied and fEPSPs were recorded. Synaptic plasticity strength was assessed by comparing the last 5 minutes of recordings after HFS. As anticipated, the control transgenic 5xFAD mice group (5xFAD + veh) had significantly decreased level of LTP formation compared to WT mice of both groups (WT + veh: 173.30% ± 17.34% and 5xFAD + veh: 144.20% ± 7.45%, *p*=0.0014; WT + hM3D: 179.30% ± 12.64% and 5xFAD + veh, *p*=0.0003; Figure 2A,B). However, repeated astrocytic modulation in 5xFAD + hM3D mice led to an enhancement of synaptic strength compared to their control transgenic littermates (5xFAD + veh: 144.20% ± 7.45% and 5xFAD + hM3D: 171.00% ± 5.10%, *p*=0.0035, all comparisons are done using two-way ANOVA with Tukey post hoc test, *F*_int_(1,21) = 5.035, *F*_row_(1,21) = 12.22, *F*_column_(1,21) = 14.67), restoring it to the WT + veh mice (*p*=0.9949). Importantly, absence of any negative impact on synaptic plasticity was observed in WT + hM3D mice group in comparison to WT + veh (*p*=0.8036; Figure 2B). Obtained results demonstrated that modulating astrocytic activity positively influences synaptic strength and LTP formation in transgenic 5xFAD mice.

**Figure 3:**
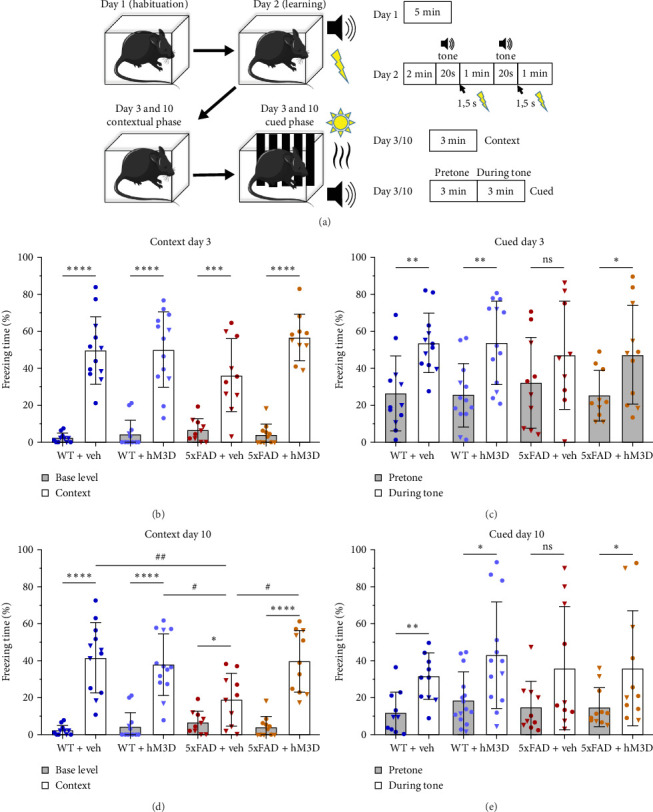
Repeated modulation of astrocytic activity beneficially impacted on 5xFAD mice memory in fear conditioning behavioral test. (A) Principal scheme of the FC experiment. (B) Total context freezing time (%) of testing groups on Day 3. (C) Total cued freezing time (%) of testing groups on Day 3. (D) Total context freezing time (%) of testing groups on Day 10. (E) Total cued freezing time (%) of testing groups on Day 10. For all comparisons, WT + veh: *n* = 12 mice (8♂ and 4♀), WT + hM3D: *n* = 13 mice (11♂ and 2♀), 5xFAD + veh: *n* = 10 mice (4♂ and 6♀), 5xFAD + hM3D: *n* = 11 mice (7♂ and 4♀). For Parts (B–D), Student's paired *t*-test for comparison with baseline level for context phase and with “pretone” for cued FC (ns: nonsignificant, *⁣*^*∗*^*p*  < 0.05, *⁣*^*∗∗*^*p*  < 0.01, *⁣*^*∗∗∗*^*p*  < 0.001, *⁣*^*∗∗∗∗*^*p*  < 0.0001); for Part (E), for comparison “pretone” and “during tone” stages Mann–Whitney test was applied (ns: nonsignificant, *⁣*^*∗*^*p*  < 0.05, *⁣*^*∗∗*^*p*  < 0.01). For multiple comparisons between mice groups two-way ANOVA with Tukey post hoc test on Part (D) (#: *p*  < 0.05, ##: *p*  < 0.01) was performed. In graphs, male mice are represented by circles and female mice by triangles. All data is presented as mean ± SD.

Further, behavioral testing was conducted to evaluate cognitive and noncognitive functions of 5xFAD mice (Figure 1B). Memory validation was carried out using FC memory testing [63–65] and Morris water [59, 66, 67] as they are closely associated with hippocampus functioning. FC was performed by classical protocol (Figure 3A). On Day 3, 24 h after learning, all groups have shown stable memory level (base level vs. context: *p*  < 0.0008, Student's paired *t*-test). Moreover, we have not detected any significantly differences between freezing levels in all groups (*p*=0.0716, two-way ANOVA following Tukey post hoc test, *F*_int_(1,41) = 3.420, *F*_row_(1,41) = 3.669, *F*_column_(1,41) = 0.405; Figure 3B). At the same time, on the associative phase of learning, 5xFAD control mice did not show any learning (pretone: 32.12% ± 24.62% vs. during tone: 47.00% ± 29.20%, *p*=0.0866, Student's paired *t*-test), while all other groups had a significantly higher response to unconditional stimulus presentation (pretone vs. during tone: *p*  < 0.0156, Student's paired *t*-test; Figure 3C).

A week later, the same procedure was held. On Day 10 at the contextual stage of FC, all groups of mice had a significantly higher level of freezing comparatively to the baseline level (base level vs. context: *p*  < 0.0202, Student's paired *t*-test). However, 5xFAD + veh mice group had a significantly poorer results than other mice groups due to an aberrant cognitive functions (context: WT + veh: 41.56% ± 18.92% and 5xFAD + veh: 18.81% ± 14.21%, *p*=0.0148; WT + hM3D: 37.82% ± 16.64% and 5xFAD + veh, *p*=0.0483; 5xFAD + veh and 5xFAD–hM3D: 39.69% ± 16.68%, *p*=0.0334, two-way ANOVA with Tukey post hoc test, *F*_int_(1,42) = 6.112, *F*_row_(1,42) = 2.962, *F*_column_(1,42) = 4.396; Figure 3D). It must be noted, that daily modulation of astrocytic activity through hM3D in WT mice did not lead to any significant changes in the contextual memory performance either on Day 3 (WT + veh: 49.72% ± 18.20% and WT + hM3D: 50.08% ± 20.36%, *p*=0.9636, Student's *t*-test) either on Day 10 (WT + veh: 41.56% ± 18.92% and WT + hM3D: 37.82% ± 16.64%, *p*=0.6046, Student's unpaired *t*-test). On the cued phase on Day 10, we have observed similar tendency as on Day 3: All mice group have shown significant increase in freezing time on tone stage vs. pretone stage (pretone vs. during tone: *p*  < 0.0281, Mann–Whitney test), except 5xFAD + veh group (pretone: 14.73% ± 13.93% vs. during tone: 35.73% ± 33.35%, *p*=0.1431, Mann–Whitney test), which did not exhibit long-term associative memory formation (Figure 3E). It should be also noted that wide distribution of 5xFAD + veh mice freezing levels in the during tone cued phase of FC testing in both Day 3 and Day 10 may underlie the lack of statistical significance between pretone and during tone phases.

**Figure 4:**
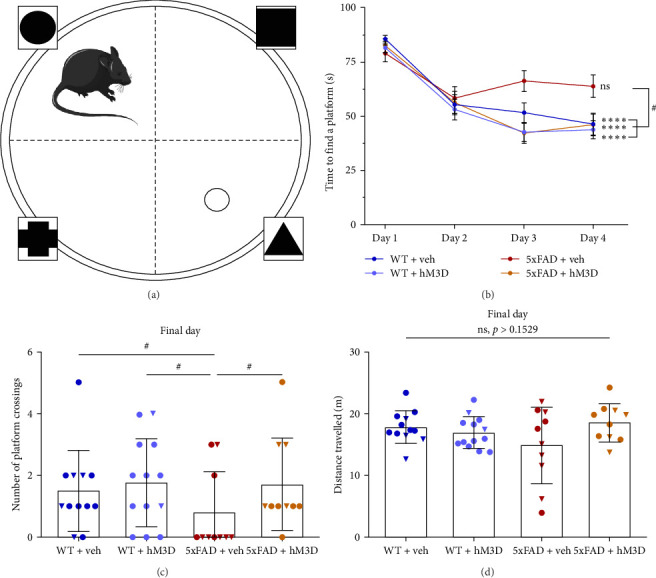
Hippocampal astrocytic activity modulation in 5xFAD mice restored abnormal spatial memory in Morris water maze behavioral test. (A) Graphical illustration of Morris water maze test. (B) Latent time as a correlate of mouse memory is disrupted in control 5xFAD + veh mice aged 8 months. (C) HM3D activation in hippocampal astrocytes led to increase in number of platform crossings on final day of MWM in 5xFAD mice. (D) No changes in mice ability to swim is detected on final day of MWM behavioral test (WT + veh: *n* = 12 mice [8♂ and 4♀]; WT + hM3D: *n* = 13 mice [11♂ and 2♀]; 5xFAD + veh: *n* = 10 mice [4♂ and 6♀], 5xFAD + hM3D: *n* = 10 mice [6♂ and 4♀]). For Part (B), comparisons between Day 1 and Day 4 of learning Kruskal–Wallis test with Dunn's test was performed (ns: no significant differences, *⁣*^*∗∗∗∗*^*p*  < 0.0001). For multiple comparisons of latent time between mice groups on Day 4 Kruskal–Wallis test with Dunn's test was used (#: *p*  < 0.05). For Part (C), Kruskal–Wallis test with Conover–Iman test was applied (#: *p*  < 0.05). For Part (D), two-way ANOVA with Tukey post hoc test was leveraged. In graphs male mice are represented by circles and female mice by triangles. Data in Part (A) is presented as mean ± SEM, while in Parts (B–D) data is presented as mean ± SD.

To confirm our findings in FC, we have performed MWM behavioral test. MWM is tightly bounded to hippocampal area condition, especially on NMDA receptors functioning (Figure 4A) [59]. In this behavioral test, memory is validated via comparing latent time to find a hidden platform and on the final day by the number of platform crossings. We have observed significant reduction of latent between first and fourth day of learning in all mice groups (WT + veh: Day 1: 85.27 ± 12.08 s and Day 4: 46.10 ± 33.72 s, *p*  < 0.0001; WT + hM3D: Day 1: 81.67 ± 19.31 s and Day 4: 43.71 ± 30.02 s, *p*  < 0.0001; 5xFAD + hM3D: Day 1: 82.33 ± 20.77 s and Day 4: 46.05 ± 33.86 s, *p*  < 0.0001, Kruskal–Wallis test with Dunn's test for multiple comparisons for all), except 5xFAD + veh mice, that did not show any significant changes in latent time (Day 1: 78.83 ± 25.27 s and Day 4: 63.63 ± 32.98 s, *p*=0.0551; Figure 4B). Moreover, while comparing latent time in the last day of learning (Day 4), significantly lesser memory was observed in the control 5xFAD + veh mice compared to other groups (WT + veh: 46.10 ± 33.72 s and 5xFAD + veh: 63.63 ± 32.98 s, *p*=0.0206; WT + hM3D: 43.71 ± 19.31 s and 5xFAD + veh, *p*=0.0204; 5xFAD + hM3D: 82.33 ± 20.77 s and 5xFAD + veh: *p*=0.0479, Kruskal–Wallis test with Dunn's test for multiple comparisons for all; Figure 4B). However, we did not define any significant difference between percent of successful trials between all mice groups in learning days (Figure S2).

On the final day, platform was removed and mice were given one 90 s trial and the number of platform crossings was tested. In accordance to above stated, number of platform crossings on final day were also significantly reduced in the 5xFAD + veh group compared to other ones (WT + veh: 1.50 ± 1.31 and 5xFAD+veh: 0.80 ± 1.32, *p*=0.0373; WT + hM3D: 1.77 ± 1.42 and 5xFAD + veh, *p*=0.0107; 5xFAD + veh and 5xFAD + hM3D: 1.70 ± 1.50, *p*=0.0281, Kruskal–Wallis test with Conover–Iman test; Figure 4C). However, the time spent in the quadrant of interest was evaluated, but no statistical differences in the metric were found. At the same time, we did not detect any influence of hippocampal astrocytic activity modulation in WT mice both on the last day learning (Day 4) (WT + veh: 46.10 ± 33.72 s and WT + hM3D: 43.71 ± 19.31 s, *p*=0.9188, Mann–Whitney test) and on final day in platform crossings number (WT + veh: 1.50 ± 1.31 and WT + hM3D: 1.77 ± 1.42, *p*=0.6039, Mann–Whitney test). To verify, whether absence of learning and significant increase of latent time on Day 4 and poor results on final day actually connected with memory alterations in control 5xFAD + veh mice group, we have checked distance traveled of all mice groups on final day. We did not find any significant differences between mice' swimming ability (*p*  > 0.153, two-way ANOVA with Tukey post hoc test, *F*_int_(1,41) = 3.957, *F*_row_(1,41) = 1.497, *F*_column_(1,41) = 0.338; Figure 4D). Consequently, MWM test results are truly corresponding to mice memory, and did not depend on their ability to swim. Thus, we demonstrated that modulating astrocytic activity via the DREAAD-Gq approach had a beneficial effect on the cognitive functions of 5xFAD mice, without any negative impact on the memory of wild-type mice, as demonstrated by FC and MWM testing (Figures 3 and 4). Moreover, LTP results strongly support behavioral tests results and confirmed consistency of the presented approach on the synaptic functioning and memory formation (Figure 2).

### 3.2. Daily Modulation of Hippocampal Astrocytic Activity Restored Decreased Anxiety Levels and Social Preference in 8-Month-Old 5xFAD Mice

**Figure 5:**
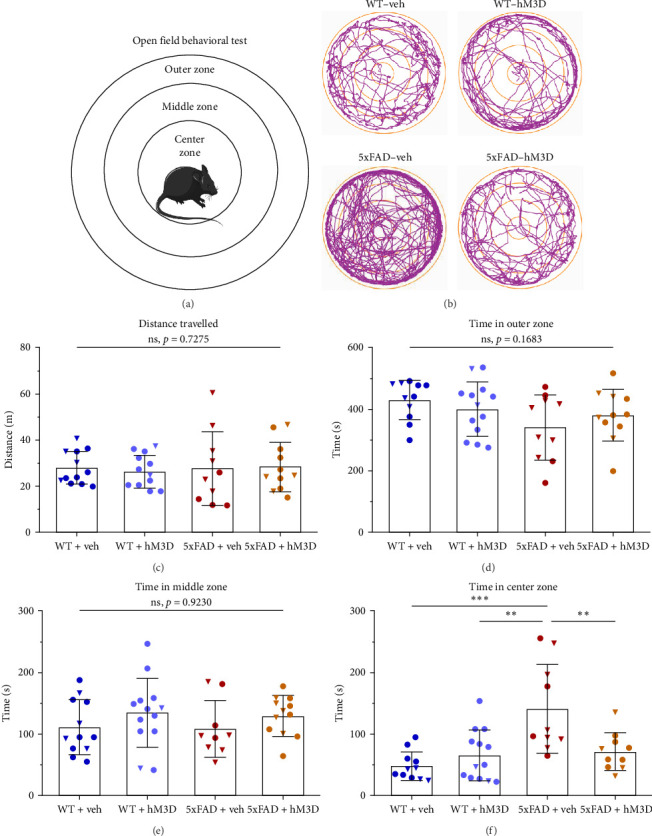
Altered anxiety levels in 5xFAD mice were rescued via repeated modulation of hippocampal astrocytic hM3D. (A) Scheme of the open filed behavioral testing. (B) Representative mice position tracking during freely behaving in open field. (C) No significant changes are observed in mice locomotion on OF testing. (D–F) Time spent on outer, middle, and center zone correspondingly. For all comparisons, WT + veh: *n* = 11 mice (7♂ and 4♀), WT + hM3D: *n* = 13 mice (11♂ and 2♀), 5xFAD + veh: *n* = 10 mice (4♂ and 6♀), 5xFAD + hM3D: *n* = 11 mice (7♂ and 4♀). Multiple comparisons are performed between mice groups using two-way ANOVA with Tukey post hoc test (*⁣*^*∗∗*^: *p*  < 0.01, *⁣*^*∗∗∗*^: *p*  < 0.001). In graphs male mice are represented by circles and female mice by triangles. All data is presented as mean ± SD.

Besides the dramatically reduced cognitive functions, AD is also accompanied by changes in noncognitive performance [68]. It is well-established that aged 5xFAD experience changes from the normal levels of anxiety and mouse-to-mouse interactions [41]. In this case, we performed an OF behavioral test to study any alterations in locomotion and anxiety. During the OF, mice were placed in the middle of the round arena and were allowed 10 min of free exploration under bright light (Figure 5A,B). First, locomotion of all mouse groups was highly similar (*p*=0.7275, two-way ANOVA with Tukey post hoc test, *F*_int_(1,42) = 0.123, *F*_row_(1,42) = 0.026, *F*_column_(1,42) = 0.080; Figure 5C), so further statements did not depend on the distance traveled by mice. Outer zone timing, near the borders of the arena, was not affected in the 5xFAD + veh group and this parameter was comparable to other groups (*p*=0.1683, two-way ANOVA with Tukey post hoc test, *F*_int_(1,42) = 1.965, *F*_row_(1,42) = 0.060, *F*_column_(1,42) = 4.709; Figure 5D). The same tendency was observed in the middle zone timing (*p*=0.9230, two-way ANOVA with Tukey post hoc test, *F*_int_(1,41) = 0.009, *F*_row_(1,41) = 2.545, *F*_column_(1,41) = 0.085; Figure 5E). However, while comparing time spent in the center zone of the arena, significantly increased time spent was found in the 5xFAD + veh mice group in comparison to all other mice (WT + veh: 47.75 ± 23.30 s and 5xFAD + veh: 140.50 ± 72.04 s, *p*=0.0002; WT + hM3D: 65.09 ± 41.43 s and 5xFAD + veh, *p*=0.0018; 5xFAD + veh and 5xFAD + hM3D: 71.26 ± 30.42 s, *p*=0.0069, two-way ANOVA with Tukey post hoc test, *F*_int_(1,42) = 9.652, *F*_row_(1,42) = 3.468, *F*_column_(1,42) = 12.60; Figure 5F). Thus, decreased anxiety levels of 5xFAD mice were totally restored after astrocytic activity modulation via daily hM3D receptors activation. Moreover, sequential hM3D-receptors activation in hippocampal astrocytes did not affect normal anxiety levels in WT mice (time in the outer zone: WT + veh: 429.70 ± 63.40 s and WT + hM3D: 400.50 ± 288.20 s, *p*=0.3699; time in the middle zone: WT + veh: 110.9 ± 44.38 s and WT + hM3D: 134.4 ± 56.04 s, *p*=0.2596; time in the center zone: WT + veh: 47.75 ± 23.30 s and WT + hM3D: 65.09 ± 41.43 s, *p*=0.2313, Student's *t*-test for all).

**Figure 6:**
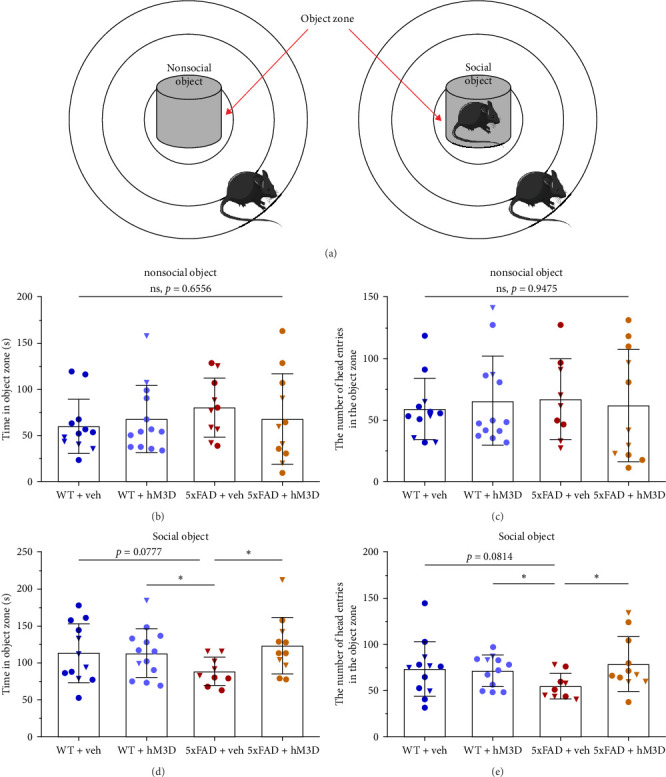
HM3D activation in hippocampal astrocytes led to restoration of normal social interest in 8-month-old 5xFAD mice. (A) Scheme of the nonsocial and social recognition behavioral tests. (B) Time spent in nonsocial object zone. (C) The number of head entries in the object zone. (D) Time spent in social object zone. (E) Amount of the head entries in the social object zone. For all comparisons, WT + veh: *n* = 12 mice (8♂ and 4♀), WT + hM3D: *n* = 12 mice (10♂ and 2♀), 5xFAD + veh: *n* = 9 mice (4♂ and 5♀), 5xFAD + hM3D: *n* = 11 mice (7♂ and 4♀). For Parts (B, C), ANOVA interaction factor is shown. Comparisons in Parts (D) and (E) between mice groups are performed by Brown–Forsythe and Welch ANOVA tests (*⁣*^*∗*^: *p*  < 0.05). In graphs male mice are represented by circles and female mice by triangles. All data is presented as mean ± SD.

Further, experiments for the investigation of the mice′ interest to nonsocial and social objects were carried out (Figure 6A,B). On the first day, nonsocial object exploration, mice were placed near the border of the arena in the outer zone and given 10 min of free movement. In this stage, we did not find any differences in exploration time (Figure 6B), and the number of head entries in the object zone (Figure 6C) was closely similar across groups (time in object zone: *F*(3,42 = 0.5430, *p*=0.6556 and the number of head entries in the object zone: *F* (3,41) = 0.1205, *p*=0.9475).

On the second day, social object exploration, the same protocol was applied, but in the middle of the arena mouse of the same sex and strain was introduced. Here, we observed a significant reduction in exploration time for 5xFAD + veh mice group (WT + veh: 113.20 ± 39.83 s and 5xFAD + veh: 88.54 ± 18.78 s, *p*=0.0777; WT + hM3D: 113.10 ± 33.01 s and 5xFAD + veh: *p*=0.0387; 5xFAD + hM3D: 123.10 ± 38.46 s and 5xFAD + veh: *p*=0.0192, Brown–Forsythe and Welch ANOVA tests with Welch correction), as well as in the number of head entries in the object zone (WT + veh: 73.00 ± 29.99 and 5xFAD + veh: 54.78 ± 13.684, *p*=0.0814; WT + hM3D: 71.00 ± 16.80 and 5xFAD + veh: *p*=0.0256; 5xFAD + hM3D: 78.55 ± 29.70 and 5xFAD + veh: *p*=0.0192, Brown–Forsythe and Welch ANOVA tests with Welch correction; Figure 6D,E). It should be noted that differences in anxiety levels could also influence the results of both the nonsocial and social preference test. In both of these behavioral tests, we did not also define any changes in WT + hM3D mice group compared to WT + veh control group (nonsocial interaction test [exploration time]: WT + veh: 60.16 ± 29.46 s and WT + hM3D: 67.99 ± 36.36 s, *p*=0.5585, Welch's test and for social interaction test [exploration time]: WT + veh: 113.20 ± 39.83 s and WT + hM3D: 113.10 ± 33.01 s, *p*=0.9976, Welch's test).

To sum up, therapeutic intervention based on modulating astrocytic activity in the hippocampus led to a significant improvement of the altered levels of anxiety in 8-month-old 5xFAD mice. Moreover, hM3D activation by CNO IP injections restored social interest in 5xFAD + hM3D transgenic mice to the normal level. Importantly, in the WT + hM3D mice group, none of these parameters were significantly impacted by the modulation of hippocampal astrocytic activity compared to their WT + veh littermates.

### 3.3. Repeated Astrocytic Activity Regulation Through hM3D Reduced Aβ-Plaques Area With a Trend Toward Decreasing Astrocytic Reactivity in the Hippocampal Area of 8-Month-Old Transgenic 5xFAD Mice

**Figure 7:**
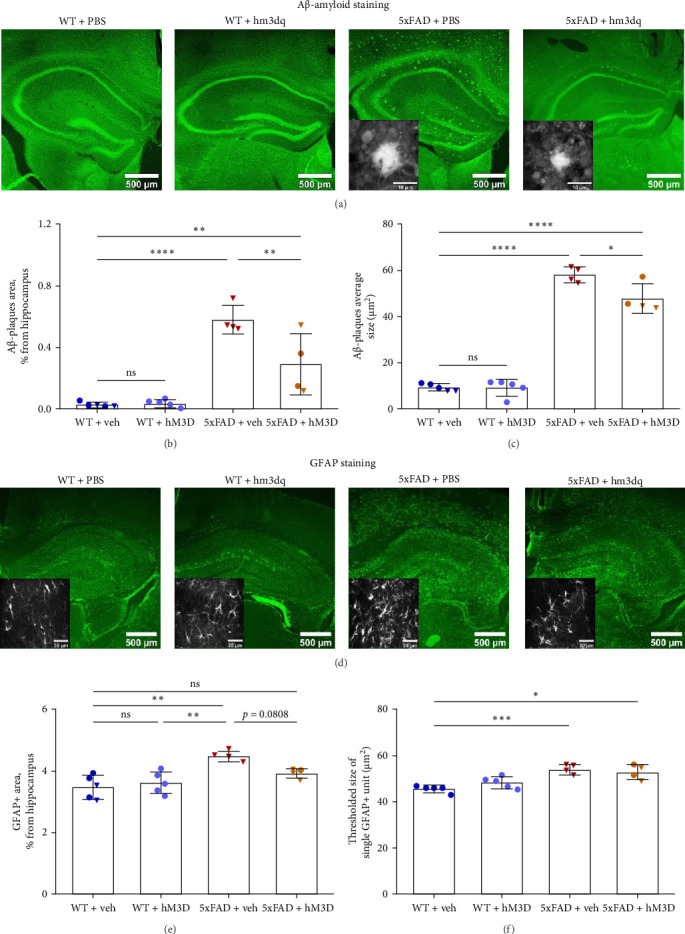
Daily modulation of hippocampal astrocytic activity through hM3D significantly reduced Aβ-amyloid plaques area with a trend toward decreasing astrocytic reactivity in 8-month-old 5xFAD mice. (A) Representative confocal images of Thioflavin-T staining in all mice groups aged 8 months. Scale bar corresponds to 500 µm (4x magnification) and to 10 µm (100x magnification). (B) Aβ-amyloid plaques area in percent of hippocampus area (%). (C) Average size of a single Aβ-amyloid plaque (µm^2^). (D) Representative confocal images of glial fibrillary acidic protein (GFAP) staining in all mice groups. Scale bar corresponds to 500 µm (4x magnification) and to 30 µm (40x magnification). (E) GFAP positive area in percent of hippocampus area. (F) Thresholded size of single GFAP positive unit (µm^2^). For comparisons in Parts (B, C, E, and F), WT + veh: *n* = 5 mice (3♂ and 2♀), WT + hM3D: *n* = 5 mice (5♂), 5xFAD + veh: *n* = 4 mice (4♀), 5xFAD + hM3D: *n* = 4 mice (2♂ and 2♀). Multiple comparisons in all graphs between mice groups are done using two-way ANOVA with Tukey post hoc test (*⁣*^*∗*^*p*  < 0.05, *⁣*^*∗∗*^*p*  < 0.01, *⁣*^*∗∗∗*^*p*  < 0.001, *⁣*^*∗∗∗∗*^*p*  < 0.0001). In graphs male mice are represented by circles and female mice by triangles. Data is presented as mean ± SD.

Toxic Aβ-amyloid plaques are believed to play a crucial role in the pathogenesis of AD [69]. Their accumulation enhances NMDA-receptor activity and imbalances intraneuronal [70] and cytosolic astrocytic calcium levels [71]. Thus, we tested whether our approach based on DREAAD (Gq) could influence Aβ-amyloid levels in the hippocampus of 8-month-old transgenic 5xFAD mice. For this purpose, we used 50 µm thick fixed sagittal slices of hippocampus and specifically visualized Aβ-amyloid plaques using Thio-T representative confocal images of all mouse groups' staining are presented in Figure 7A. Some nonspecific binding of Thio-T was observed, resulting in low levels of Aβ-amyloid-positive area detected in the WT + veh and WT + hM3D mouse groups. The analysis was conducted across the entire hippocampal formation (CA1, CA2, CA3, hilus, and dentate gyrus). As anticipated, we observed a highly dense accumulation of Aβ-amyloid in the hippocampal area of transgenic 5xFAD mice (WT + veh: 0.026% ± 0.017% and 5xFAD + veh: 0.579% ± 0.093%, *p*  < 0.0001; Figure 7 B). Moreover, we detected a significant reduction in amyloid plaques area in the 5xFAD + hM3D group (5xFAD + veh: 0.579% ± 0.093% and 5xFAD + hM3D: 0.290% ± 0.199%, *p*=0.0067). However, hM3D activation did not lead to complete clearance of Aβ-amyloid from the hippocampus (WT + veh: 0.026% ± 0.017% and 5xFAD + hM3D: 0.290% ± 0.199%, *p*=0.0087, Figure 7B). It must be also noted that daily astrocytic hM3D receptor activation reduced the average plaque area in 5xFAD-mice (5xFAD + veh: 57.90 ± 3.37 µm^2^ and 5xFAD + hM3D: 47.63 ± 6.39 µm^2^, *p*=0.0119, two-way ANOVA with Tukey post hoc test for all presented comparisons of Aβ-amyloid average plaque size, *F*_int_(1,14) = 7.276, *F*_row_(1,14) = 7.708, *F*_column_(1,14) = 535.000; Figure 7C). Moreover, hippocampal astrocytic activity modulation was not amyloidogenic (WT + veh: 0.026% ± 0.017% and WT + hM3D: 0.036% ± 0.024%, *p*=0.9995, two-way ANOVA with Tukey post hoc test for all presented comparisons of Aβ-amyloid plaques area, *F*_int_(1,14) = 9.408, *F*_row_(1,14) = 8.826, *F*_column_(1,14) = 68.61).

Further, to investigate the influence of hM3D activation in mouse hippocampus on astrocytes status and reactivity, we carried out immunohistochemical staining for the GFAP (Figure 7 D). In AD, astrocytes transform into reactive ones and increased GFAP expression is a marker of such reactivity. Indeed, in 8-month-old 5xFAD + veh transgenic mice, we have detected elevated levels of GFAP positive area in the hippocampus (WT + veh: 3.471% ± 0.393% and 5xFAD + veh: 4.474% ± 0.166%, *p*=0.0011; Figure 7E). Daily astrocytes hM3D activation demonstrated a trend toward decreasing GFAP levels in hippocampal area of transgenic 5xFAD mice (5xFAD + veh: 4.474% ± 0.166% and 5xFAD + hM3D: 3.910% ± 0.157%, *p*=0.0808; Figure 7 E). Importantly, astrocytic activity modulation did not induce reactive transformation in the wild type mice (WT + veh: 3.471% ± 0.393% vs. WT + hM3D: 3.607% ± 0.354%, *p*=0.8907, two-way ANOVA with Tukey post hoc test, *F*_int_(1,14) = 5.968, *F*_row_(1,14) = 2.231, *F*_column_(1,14) = 20.770; Figure 7 E). Moreover, we detected an increase in the thresholded size of single GFAP + unit in control transgenic 5xFAD mice (WT + veh: 45.66 ± 1.54 µm^2^ and 5xFAD + veh: 53.94 ± 2.24 µm^2^, *p*=0.0008, two-way ANOVA with Tukey post hoc test, *F*_int_(1,14) = 2.908, *F*_row_(1,14) = 0.4145, *F*_column_(1,14) = 30.23; Figure 7 F. Observable trend toward ameliorating astrocytic reactivity in the 5xFAD + hM3D group might be attributed to the clearance of toxic Aβ-amyloid from the hippocampus.

## 4. Discussion

Over the past decades, the brain has increasingly been understood as a network of coordinated activity among all its cells, including neurons, astrocytes, and microglia [72]. Astrocytes form a tripartite synapse with the pre- and postsynaptic membranes of neurons, regulating a wide range of functions such as modulating synaptic transmission, absorbing and recycling neurotransmitters, and releasing co-agonists of neuronal receptors and gliotransmitters [23, 73–75]. Thus, achieving selective control of astrocytes under pathological conditions is crucial for developing novel therapeutic approaches [30, 76]. In AD, astrocytes transition into a reactive state, characterized by increased size and the secretion of proinflammatory factors [29]. They also lose their normal functional capacity, disrupting neuronal signaling, and plasticity. In this context, modulating astrocytic activity could be essential for restoring their function and regulating neurotransmitter release, and, consequently, normalizing normal neuronal functioning [30, 31, 77, 78].

Modulating astrocytic activity has emerged as a promising strategy for counteracting pathological changes associated with various neurodegenerative disorders, particularly AD. Numerous studies have demonstrated the beneficial effects of astrocyte-targeted interventions on restoring hippocampal and broader brain functions in AD mouse models [30]. On the one hand, enhancing astrocytic function can yield therapeutic benefits. For instance, expression of channelrhodopsin-2 (ChR2) in hippocampal astrocytes restored slow brain rhythms and decreased Aβ-amyloid plaques deposition in APP/PS1 mice [77]. Similarly, optogenetic stimulation of cultured astrocytes expressing ChR2 under Aβ-amyloid toxicity conditions was able to restore dysregulated astrocytic calcium signaling [78]. On the other hand, suppressing astrocytic expression of pro-inflammatory or disease-promoting proteins represents another viable therapeutic approach. For example, knockout of the astrocytic glycoprotein YKL-40 reduced amyloid burden and improved memory performance in 5xFAD mice [79]. Likewise, silencing P2Y1 receptors in astrocytes attenuated neuroinflammation and cognitive deficits in APP/PS1 mice [69]. Aligned with abovementioned, we recently demonstrated that optogenetic activation of Opto-α1AR (a Gq-coupled receptor) expressed in hippocampal astrocytes can rescue LTP formation and cognitive function in 6-month-old 5xFAD mice [34]. However, this approach lacks translational potential due to the requirement for fiber implantation for light delivery and the localized nature of astrocytic modulation. To address this limitation, we employed a chemogenetic approach in this study, using DREADDs receptors—specifically hM3D(Gq) under the GFAP astrocytic promoter—to control astrocytic activity in the hippocampus [38, 80]. It is worth mentioning that the hippocampus is one of the brain's neurogenic niches [81], and therefore, hM3D may also be expressed in some differentiating progenitor cells, however, presumably, their contribution to the observed results is minimal. These receptors are selectively activated by CNO, which has no natural targets in the mammalian organism [82]. Unlike optogenetics, this method enables widespread modulation of astrocytes within the targeted region rather than being restricted to local stimulation [83]. To assess the effectiveness of astrocytic modulation, we bilaterally expressed hM3D(Gq) in the hippocampus of both experimental wild-type and transgenic 5xFAD mice, while control mice received bilateral vehicle injections. Receptor activation was achieved through daily IP injections of CNO (3 mg/kg).

AD is characterized by a severe decline in cognitive function. These deficits are also observed in various AD mouse models, including 6-month-old and older 5xFAD mice. Behavioral assessments are crucial for studying cognitive impairments, with the FC and MWM tests being particularly relevant due to their strong dependence on hippocampal function [63, 67]. We observed that repeated activation of hM3D(Gq) in astrocytes had a beneficial effect on memory retention in the contextual phase of FC testing in transgenic 5xFAD mice. In contrast, control 5xFAD mice exhibited a significant decline in contextual memory, especially 1 week after learning. A similar trend was observed in the MWM test, where control 5xFAD mice demonstrated an absence of learning and cognitive decline was correlated to a significantly increased latency to find the platform compared to the WT + veh, WT + hM3D, and 5xFAD + hM3D groups. Moreover, cognitive deficits were also evident on the final day of MWM testing, with control 5xFAD mice showing a significantly reduced ability to cross the platform. Importantly, these cognitive impairments were independent of swimming ability, as all groups displayed similar average swimming speeds. It should be noted that in our previous study, which relied on optogenetic stimulation of Gq-coupled signaling in astrocytes [34], mice failed to achieve stable memory improvement due to limitations associated with fiber implantation. In contrast, the current work builds on this knowledge and clearly demonstrates the advantages of the chemogenetic approach over the optogenetic method in investigating the effects of astrocytic Gq-coupled receptors activation on cognitive function in an AD mouse model. Notably, astrocytic modulation in wild-type mice did not influence normal memory formation in either cognitive test. This suggests that astrocytic function in the healthy brain is already optimized, and external repeated modulation may not further enhance memory or cognitive performance. Likewise, the absence of memory enhancement might be attributed to the lack of precise temporal control over astrocytic activity. However, previous studies have shown that specific in time targeted optogenetic stimulation of astrocytes can enhance memory performance in mice [25]. Nevertheless, it remains unclear whether the effects observed following astrocytic stimulation are due to enhancements in learning, memory, or whether improved learning secondarily facilitates better memory retention [84]. To shed light on this important question, future studies are needed to dissect the specific contributions of astrocytic activity modulation—using tools such as ChR2 or OptoXRs—on learning and memory formation. Behavioral paradigms incorporating reversal learning tasks may be particularly valuable, as they can help distinguish between facilitation of learning and genuine memory enhancement [85, 86]. Moreover, our findings highlight the crucial role of astrocytic modulation in restoring synaptic plasticity deficits associated with AD. Daily hM3D activation expressed in the hippocampal astrocytes effectively enhanced synaptic strength in transgenic 5xFAD mice, bringing LTP formation to wild-type levels without adverse effects on healthy controls. These results reinforce the link between astrocytic function and synaptic integrity, aligning with behavioral improvements observed.

The beneficial impact of sequential astrocytic modulation on cognitive function and synaptic plasticity can be explained from several perspectives. On the one hand, modulation of Ca^2+^ signaling in hippocampal astrocytes triggers the release of a broad range of gliotransmitters, such as glutamate, adenosine, and D-serine [33, 87, 88]. Glutamate release may activate NMDA or AMPA receptors, thereby enhancing various forms of long-term plasticity [89]. However, NMDA receptor upregulation is a well-known hallmark of AD, often contributing to neuronal hyperactivity and excitotoxicity. Therefore, the observed beneficial effects of astrocytic activity in the hippocampus on cognitive function and plasticity are only weakly associated with glutamate release [90, 91]. Concurrently, our recent findings have shown that optogenetic stimulation of Opto-α1AR in hippocampal astrocytes leads to an upregulation of EAAT-2 receptor expression. EAAT-2 plays a critical role in glutamate uptake, which may help prevent excitotoxicity and is likely correlated with improved cognitive function and synaptic plasticity [92]. Moreover, adenosine released from astrocytes following astrocytic activation (as was shown by optogenetic activation of astrocytes in hippocampus [93]) can stimulate adenosine receptor type 1 (A1R), which is essential for regulating neuronal excitability [94, 95]. While A1R expression is known to be downregulated in AD, its role in maintaining excitatory–inhibitory (E–I) balance remains crucial. Thus, astrocytic adenosine release could activate the remaining A1Rs, thereby restoring E–I balance and contributing to normalized brain function. Additionally, studies underscore the essential role of D-serine in the formation of long-term potentiation and overall cognitive function [96, 97]. Activation of hM3D in hippocampal astrocytes promotes the release of D-serine [25], which facilitates memory formation and cognitive performance by modulating NMDA receptor activity [15, 98]. In AD, D-serine levels are disrupted, as demonstrated in APP-KO mice, where intracellular D-serine is elevated while extracellular levels are reduced [99]. Moreover, impairment of glycolysis-derived L-serine production in astrocytes contributes to cognitive deficits in AD [100]. Modulation of astrocytic Ca^2+^ signaling may help normalize D-serine production and release, ultimately supporting the restoration of plasticity mechanisms and cognitive function. On the other hand, AD is characterized by significant alterations in brain metabolism, particularly a reduction in hippocampal glucose uptake [101, 102]. This metabolic dysfunction results in decreased intracellular pyruvate and ATP levels, impairing the function of brain cells. Glucose uptake is vital for maintaining cognitive performance and synaptic plasticity [103]. This notion is further supported by a recent study [104], in which the authors demonstrated enhanced hippocampal recovery via restored glucose metabolism following hM3D activation in astrocytes within a kainic acid-induced neurodegeneration model.

Beyond cognitive deficits, AD is also associated with alterations in noncognitive parameters, such as anxiety [105, 106]. To investigate this, we conducted an OF test followed by nonsocial and social recognition behavioral tests. Control 5xFAD + veh mice exhibited significantly reduced anxiety levels [107], which were fully restored to WT levels following astrocytic modulation with hM3D. Additionally, control 5xFAD mice displayed reduced social interest, which was also rescued by daily Gq-coupled signaling activation. Importantly, no significant negative effects of astrocytic modulation were observed in wild type mice across these behavioral tests. These noncognitive tests are highly sensitive to the general condition of the mice; therefore, effectively assessing the impact of endogenous astrocytic Gq-coupled receptor activation on noncognitive functions is best achieved using the chemogenetic approach, given the highly invasive nature of optogenetics. The hippocampal region is involved in a wide range of higher-order neural functions, including emotional regulation [108–110]. In 8-month-old 5xFAD mice, impaired glutamate uptake, excitotoxicity, and disrupted E–I balance within hippocampal neuronal circuits may contribute to significant alterations in anxiety levels and social behavior. The restoration of these functions—potentially mediated by adenosine release from astrocytes [93, 111] and the reestablishment of normal E–I balance may underlie the beneficial effects of modulation of hippocampal astrocytic activity on noncognitive performance.

A hallmark of AD is the accumulation of toxic Aβ-amyloid plaques [2], which disrupt normal calcium signaling and induce neuronal hyperactivation and death. Aβ-amyloid pathology also negatively affects astrocytes, driving them into a reactive state [112, 113]. Immunohistochemical analysis revealed a positive effect of hippocampal astrocytic modulation, with a significant reduction in both the number and size of Aβ-amyloid plaques in 5xFAD + hM3D mice compared to control 5xFAD + veh mice. GFAP labeling further revealed a significant increase in astrocyte reactivity in the hippocampus of control 5xFAD + veh mice compared to all other groups. However, hM3D activation effectively reduced Aβ-amyloid plaque area in 5xFAD + hM3D mice with a trend towards decreasing astrocyte reactivity. Despite this reduction, Aβ-amyloid deposition remained elevated in experimental 5xFAD mice compared to WT controls. Nevertheless, the observed decrease in Aβ-amyloid burden is a promising outcome, suggesting that hippocampal astrocytic modulation may help mitigate pathological processes in AD. The observed reduction in hippocampal Aβ-amyloid plaque area is consistent with a previous study, in which astrocytic modulation using hM3D also resulted in decreased Aβ42 levels in the hippocampus [114]. Importantly, repeated astrocytic modulation did not alter GFAP levels in WT + hM3D mice, indicating that astrocytes were not transformed into a reactive state. The trend toward reduction of astrocytic reactivity observed in the 5xFAD + hM3D mice group may be attributed to the reprogramming of hM3D-expressing astrocytes toward a homeostatic or neuroprotective phenotype [29, 71, 115]. This scenario could account for observed results in GFAP-positive area in the hippocampus of 5xFAD + hM3D mice, despite the absence of changes in the average area of individual astrocytes within the hippocampus of the 5xFAD + hM3D mice group. Astrocytes are known to secrete a wide array of anti-inflammatory and antiamyloidogenic factors, including brain-derived neurotrophic factor (BDNF)—which has been shown to be released by astrocytes following calcium release-activated channels (CRACs) signaling activation via Opto-STIM1 in the cortex [116]—as well as other neurotrophic molecules, interleukin(IL)-6 [117], IL-10 [118], and thrombospondins [119, 120]. Additionally, the potential normalization of aquaporin expression, particularly AQP4, in astrocytes may contribute to enhanced Aβ-amyloid clearance and a reduction in neuroinflammation [121].

In recent years, chemogenetic approaches have emerged as a promising tool for modulating astrocytic activity to restore neuronal functioning under pathological conditions [80, 122, 123]. The beneficial effects of Gq-pathway activation in astrocytes have been demonstrated in a stroke model, where Opto-STIM1 activation in cortical astrocytes promoted recovery via BDNF secretion and neuronal activation [116]. Similarly, in kainic acid-induced hippocampal neurodegeneration, astrocytic hM3D activation facilitated hippocampal recovery through glucose metabolism restoration [104]. Our findings align with these studies, demonstrating that activation of astrocytic hM3D improves cognitive function and astrocyte homeostasis under pathological conditions. Further investigation into the underlying mechanisms of astrocytic modulation in AD could uncover novel therapeutic strategies for neurodegeneration. Additionally, the development of a precise, noninvasive method for controlling Ca^2+^ signaling in astrocytes in a hM3D(Gq) manner may offer a potential approach for mitigating hippocampal neurodegeneration in AD.

## 5. Conclusion

Our study highlights the potential of chemogenetic modulation of hippocampal astrocytes as a possible therapeutic strategy for AD. By activating hM3D(Gq) receptors in hippocampal astrocytes synaptic plasticity was restored and cognitive function in 5xFAD mice were improved without adverse effects on wild type controls. Additionally, daily astrocytic modulation alleviated noncognitive deficits, such as anxiety and social impairments. Importantly, we observed a significant reduction in Aβ-amyloid plaques accumulation with a trend toward mitigating astrocytes' reactivity in the hippocampal area, indicating a potential role in mitigating pathological processes in AD. These findings align with previous studies demonstrating the neuroprotective effects of astrocytic modulation in various neurodegenerative conditions. However, further research is needed to optimize noninvasive techniques for astrocyte modulation and to explore the precise molecular mechanisms involved. Ultimately, our results contribute to the growing body of evidence supporting astrocytes as key targets for neurodegenerative disease therapies.

## Data Availability

The data that support the findings of this study are available from the corresponding author upon reasonable request.

## Supporting Information

Supporting Information Additional details on the protocol for GFAP+ cell analysis, as well as supplementary statistical evaluations of the MWM behavioral test, are available in the Supporting Information section.
