# Supplementary material for: Chemogenetic Modulation of Astrocytic Activity Rescues Hippocampus Associated Neurodegeneration in Alzheimer's Disease Mice Model 5xFAD
Source: Neural Plast. 2025 Oct 6;2025:9880933. doi: 10.1155/np/9880933 (PMC12517986; doi:10.1155/np/9880933)
Supplement: Supporting Information — Additional details on the protocol for GFAP+ cell analysis, as well as supplementary statistical evaluations of the MWM behavioral test, are available in the Supporting Information section. [file 9880933.f1.docx]

***Supplementary materials***


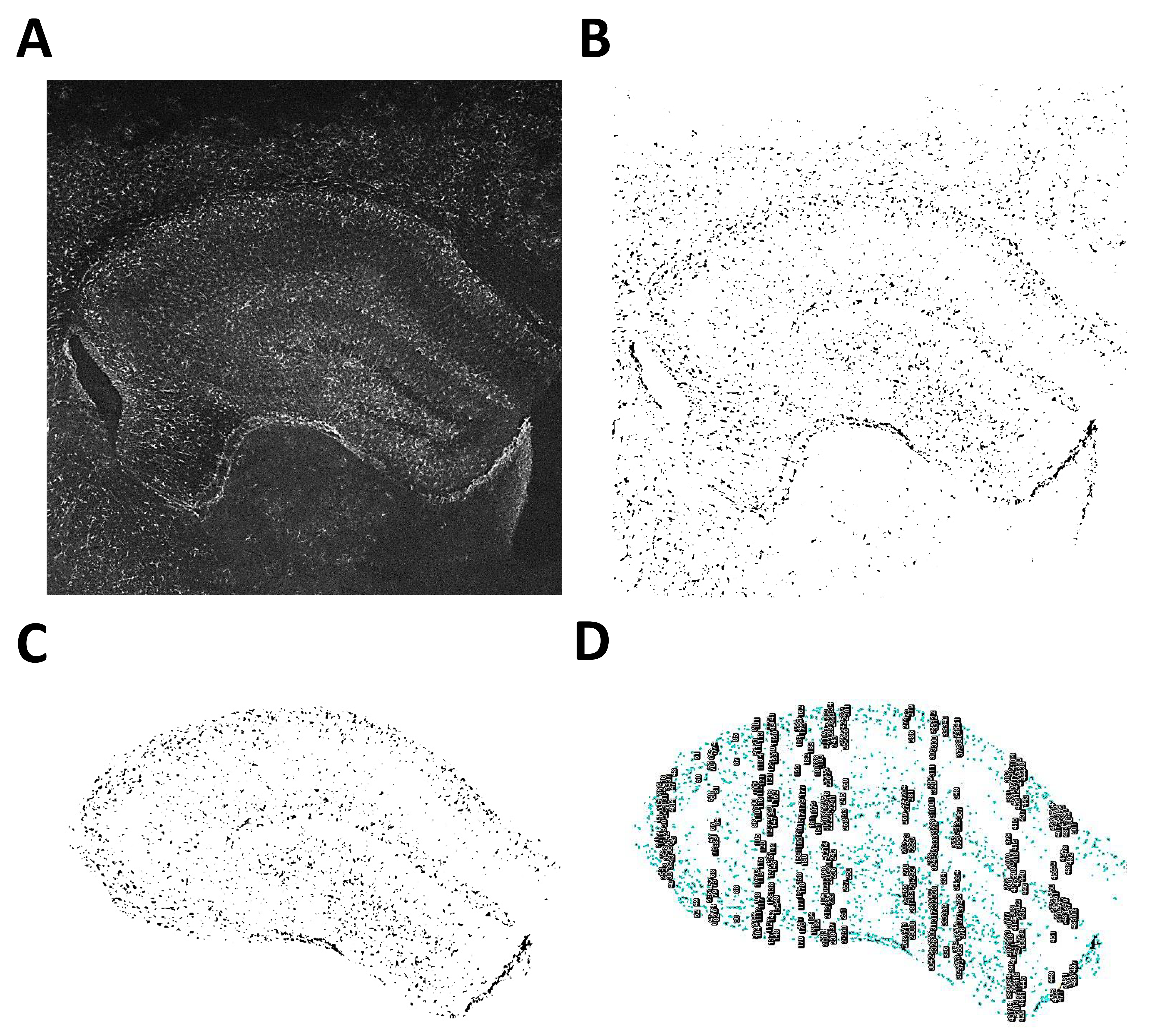


**Figure S1. GFAP positive area and thresholded size of single GFAP positive unit analysis**

(A) Confocal z-stack image of GFAP immunostaining (4x magnification). (B) Binarized image of thresholded GFAP+ units. (C) Thresholded GFAP positive units in the selected hippocampal region (CA1, CA2, CA3, hilus, dentate gyrus). (D) Several marked thresholded single GFAP+ units in the hippocampal region (total amount 2115).





**Figure S2. Percent of successful trials in Morris water maze test were closely the same between all mice groups.** (A) Percent of successful trials at Day 1 of learning. (B) Percent of successful trials at Day 2 of learning. (C) Percent of successful trials at Day 3 of learning. (D) Percent of successful trials at Day 4 of learning. WT+veh: n=12 mice (8♂ and 4♀); WT+hM3D: n=13 mice (11♂ and 2♀); 5xFAD+veh: n=10 mice (4♂ and 6♀) and 5xFAD+hM3D: n=10 mice (7♂ and 4♀)). For all comparisons Kruskal-Wallis test with Dunn’s test was applied. In graphs male mice are represented by circles and female mice by triangles. All data is presented as mean ± SD.
